# Seeding, Plating and Electrical Characterization of Gold Nanowires Formed on Self-Assembled DNA Nanotubes

**DOI:** 10.3390/molecules25204817

**Published:** 2020-10-20

**Authors:** Dulashani R. Ranasinghe, Basu R. Aryal, Tyler R. Westover, Sisi Jia, Robert C. Davis, John N. Harb, Rebecca Schulman, Adam T. Woolley

**Affiliations:** 1 Department of Chemistry and Biochemistry, Brigham Young University, Provo, UT 84602, USA; dulashani13@gmail.com (D.R.R.); aryalbasu99@gmail.com (B.R.A.); 2 Department of Physics and Astronomy, Brigham Young University, Provo, UT 84602, USA; tyler.westover13@gmail.com (T.R.W.); davis@byu.edu (R.C.D.); 3 Johns Hopkins Institute for Nanobiotechnology, Johns Hopkins University, Baltimore, MD 21218, USA; jiasisi1208@outlook.com (S.J.); rschulm3@jhu.edu (R.S.); 4 Department of Chemical Engineering, Brigham Young University, Provo, UT 84602, USA; john_harb@byu.edu

**Keywords:** DNA-templated nanofabrication, current-voltage curve, nanomaterials, resistivity

## Abstract

Self-assembly nanofabrication is increasingly appealing in complex nanostructures, as it requires fewer materials and has potential to reduce feature sizes. The use of DNA to control nanoscale and microscale features is promising but not fully developed. In this work, we study self-assembled DNA nanotubes to fabricate gold nanowires for use as interconnects in future nanoelectronic devices. We evaluate two approaches for seeding, gold and palladium, both using gold electroless plating to connect the seeds. These gold nanowires are characterized electrically utilizing electron beam induced deposition of tungsten and four-point probe techniques. Measured resistivity values for 15 successfully studied wires are between 9.3 × 10^−6^ and 1.2 × 10^−3^ Ωm. Our work yields new insights into reproducible formation and characterization of metal nanowires on DNA nanotubes, making them promising templates for future nanowires in complex electronic circuitry.

## 1. Introduction

Nanofabrication, which is used to construct structures and devices with minimum dimensions below 100 nm [1], is having a significant impact on diverse areas, such as electronics, biomedicine, materials and so forth [2]. Most current nanofabrication relies on top-down technology [3], which is highly automated but also expensive and requires complicated instrumentation [4]. Thus, nanofabrication could benefit from alternative techniques, including bottom-up approaches wherein chemical or physical forces operating at the nanoscale assemble smaller parts into larger structures [5]. Bottom-up methods produce less waste, utilize less expensive tools and offer straightforward scaling up compared to top-down nanofabrication [1,3]. Examples of bottom-up techniques include molecular self-assembly and atomic layer deposition [6].

DNA is one of the best studied bottom-up nanofabrication systems. The concept of using DNA as a nanoscale building material, though prevalent in Nature, was first explored experimentally by Nadrian Seeman in the 1980s [7]. In 2006, Rothemund demonstrated that DNA nanostructures with well-defined shapes could be constructed by repeated folding of a long, single-stranded DNA (scaffold) with hundreds of short, synthetic, single-stranded (staple) DNAs to create 2-D objects about 100 nanometer in size called DNA origami [8]. Self-assembled DNA nanotubes [9], formed using tiles, DNA origami or multiple rungs, can be created either with or without defined lengths. DNA nanotubes are further versatile because they can be functionalized using their designed DNA sequence [10] and have high surface-to-volume ratios to build large structures [11]. DNA nanotubes have emerged in DNA nanotechnology [12] as a means for creating varied structures that can be used, for example, as templates [13] or in cargo delivery [14]. These DNA nanotubes are advantageously more rigid, highly charged for electrostatic material localization and resistant to enzymatic degradation than simple single- or double-stranded DNA.

Controlled placement of DNA nanostructures on surfaces at designed locations remains a challenging problem. Gopinath et al. [15] achieved the electrostatic self-assembly of DNA origami onto lithographically defined binding sites on Si/SiO_2_ but this approach still required high resolution photolithography and might not be able to orient structures like DNA nanotubes into contiguous circuit arrangements. Shetty et al. [16] demonstrated a cleanroom-free DNA origami placement method using nanosphere lithography with a maximum yield of 74%. Rothemund and coworkers [17] also showed DNA origami alignment on SiO_2_ with tight angular precision by engineering the energy landscape of DNA origami shapes on binding sites. DNA nanotubes can be made to grow from specific locations, allowing control of nanotube positioning and reducing defects [18]. These DNA nanotubes can also join end to end to form stable connections [19] with different separation distances and relative orientation. The ability to connect between specified surface locations could be especially powerful for self-assembling nanoelectronics and crossed nanowire memory storage [20].

DNA is not electrically conductive over hundreds of nanometers, so functionalization is necessary for creating conductive wires [21]. Metallization of DNA structures typically involves binding of seed nanomaterials, followed by electrochemical growth. The first nanoscale DNA metallization was done by Braun et al. [22], who performed silver ion metallization on λ-DNA. Subsequent work has expanded to different metals including palladium [23], platinum [24], nickel [25], copper [26] and gold [27]. DNA nanostructures were also used as molds to control the growth of finite-sized metal nanostructures [28,29,30,31]. Furthermore, finite structures created by selective metallization schemes [32] and DNA structure-assisted lithography [33] showed promising progress for nanoscale DNA-assisted metallization. The best way to confirm successful metallization is to measure nanowire resistance or conductance. Two-terminal current-voltage (I-V) measurements on a DNA templated silver nanowire yielded a resistance of 200 Ω, corresponding to a resistivity of 2.4 × 10^−6^ Ωm [34]. Park et al. [35] created 1-D silver nanowires using three-helix bundle DNA tiles and performed electrical characterization on ~400 nm long wires. The I-V curves showed mostly linear behavior and gave resistances of 1.42 kΩ and 1.21 kΩ for two separate nanowire sections with 430 and 320 nm electrode gap lengths measured at 0.1 V, corresponding to bulk resistivities of 2.24 × 10^−6^ and 2.57 × 10^−6^ Ωm, respectively [35]. Liu et al. [36] demonstrated the use of self-assembled DNA nanotubes to form conductive silver nanowires; they reported two-terminal I-V curves with mostly ohmic behavior for electrode gap distances of 180, 80 and 100 nm, with resistances of 2.80, 2.35 and 2.82 kΩ at 0.1 V, corresponding to resistivities of ~1.4 × 10^−5^, 3.2 × 10^−5^ and 3.1 × 10^−5^ Ωm, respectively [36]. The resistivity of polycrystalline silver is more than 140 times smaller (1.6 × 10^−8^ Ωm) than any of the above-mentioned silver nanowires on DNA nanotubes. These previous successful I-V measurements were also done for only three or fewer nanowires and at a specific voltage. Thus, improvements are needed for producing long, metallized nanowires and obtaining multiple I-V measurements.

**Figure 1:**
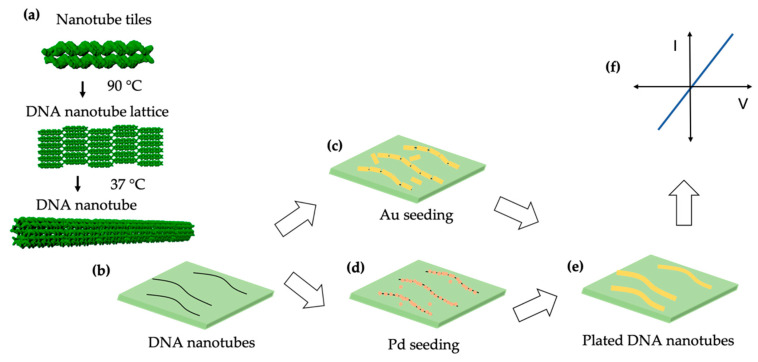
Schematic overview. (**a**) DNA nanotube assembly. (**b**) DNA nanotubes on an oxidized Si wafer. DNA nanotubes seeded with (**c**) Au or (**d**) Pd. (**e**) Plated DNA nanotubes. (**f**) I-V measurement.

Here, we present a method for metallizing DNA nanotubes to form conductive nanowires that are much longer (up to ~2 µm) than previously reported. Figure 1 shows a schematic overview of the sample preparation and characterization process. In panel (a) assembly of DNA nanotubes from tiles consisting of five DNA strands is depicted and Scheme S1 shows the tile sequences. Next, those DNA nanotubes are deposited onto an oxidized Si wafer (Figure 1b). Figure 1c,d shows seeding, either with Au nanorods or Pd ions that assemble on the DNA nanotubes. As shown in Figure 1e the plating of DNA nanotubes with Au results in nanowires, that are then probed electrically by four-point measurement in Figure 1f. This work is novel because we report the first successful four-point probe measurements on 15 different metallized DNA nanotube nanowires. Nearly 40% of the nanowires connected by electron beam induced deposition (EBID) yielded four-point I-V results with scanning between −1 and +1 V. The DNA nanotubes presented in this study have the ability to self-assemble from one end to another [19], which in the future should allow reliable fabrication of interconnects in nanoscale electronic and sensing devices.

## 2. Results

### 2.1. Deposition of DNA Nanotubes 

**Figure 2:**
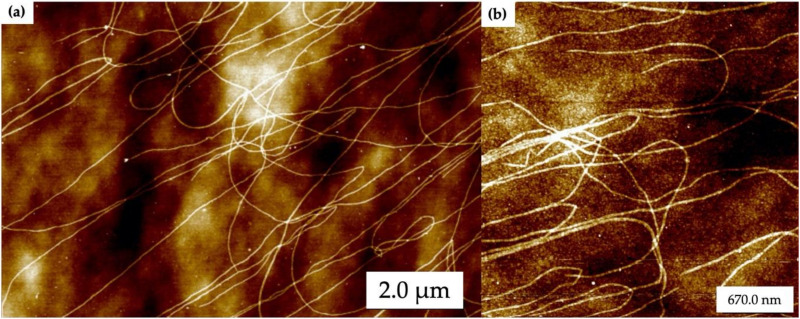
Atomic force microscopy (AFM) images of self-assembled DNA nanotubes. (**a**) Large area and (**b**) zoomed in images. Height scale is 8 nm.

Figure 2 shows atomic force microscopy (AFM) images of self-assembled DNA nanotubes that have been deposited on oxidized Si wafers. Typically, the DNA nanotubes were 1 to 10 µm in length with widths around 30 nm. In Figure 2 the random landing of DNA nanotubes on the oxidized Si surface can be seen, as well as some nanotube entanglement. Figure 2b shows a closer view of the DNA nanotubes. These results demonstrate that the DNA nanotubes were well-formed during the annealing process.

### 2.2. Au Nanorod Seeding of DNA Nanotubes

**Figure 3:**
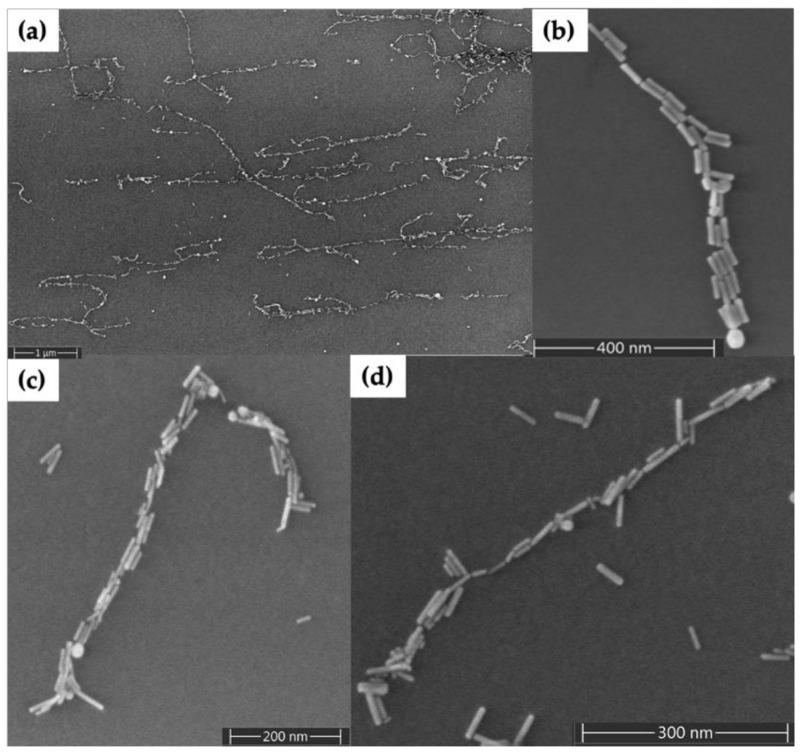
Scanning electron microscopy (SEM) images of Au nanorod-seeded DNA nanotubes. (**a**) Large area view. (**b**–**d**) Zoom views of individual DNA nanotubes seeded with Au nanorods.

Figure 3 shows results for seeding of Au nanorods on DNA nanotubes randomly deposited on the surface. Figure 3a shows a large area scanning electron microscopy (SEM) image of Au nanorod-seeded DNA nanotubes; several Au nanorod-seeded structures appear to be continuous for several micrometers in length, whereas others extend only over the hundred-nanometer scale. Just a few Au nanorods have crossed each other during seeding, because they were attracted electrostatically to the deposited DNA nanotubes. A small number of Au nanorods not associated with DNA nanotubes remained on the surface after the washing step (surfaces are difficult to image before the washing step). Overall, the relatively few Au nanorods on the surface that were not associated with DNA nanotubes indicate a clean background using our methods. Figure 3b–d shows individual Au nanorod-seeded structures. Gaps of a few nanometers are observed along every few hundred nanometers of the DNA nanotubes in these zoomed in images; as one example, see Figure 3c near the top. Well-aligned Au nanorods along the length direction of the DNA nanotubes and multiple rods in parallel can be seen in these seeded structures. Furthermore, very few spherical Au nanoparticles (nanorod synthesis impurities) are present on the Au nanorod-seeded structures. Figure S1 shows an AFM height image and corresponding section analysis for Au nanorod-seeded DNA nanotubes. The DNA nanotube heights in areas without nanorods were measured to be 2.5 ± 0.7 nm (*n* = 20), and the Au nanorod heights were determined to be 7.3 ± 1.1 nm (*n* = 20).

### 2.3. Au Nanorod-Seeded and Au-Plated DNA Nanotubes

**Figure 4:**
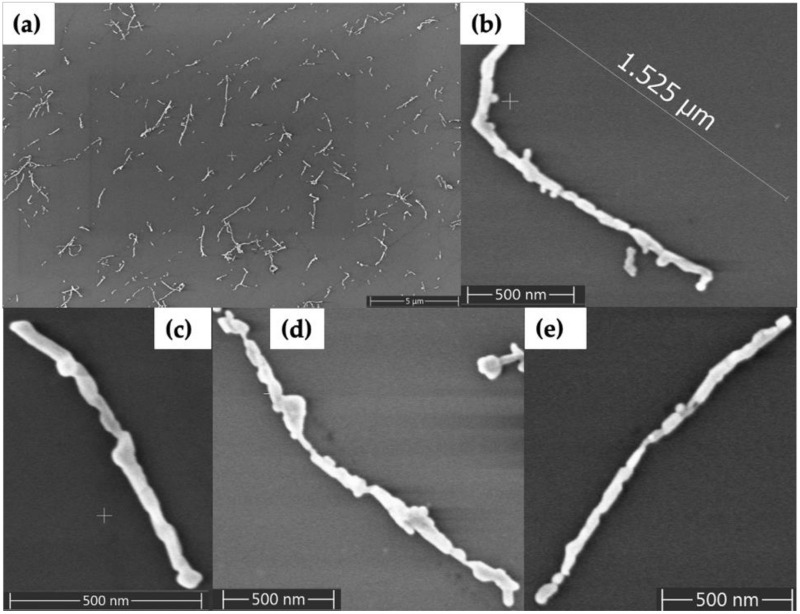
SEM images of Au nanorod-seeded and Au-plated DNA nanotubes. (**a**) Large area view. (**b**–**e**) Zoom views of individual plated DNA nanotubes.

In order to fill in the gaps between Au nanorods and form continuous metal nanowires, we used electroless plating. Figure 4 shows Au nanorod-seeded structures after the electroless Au plating process. Figure 4a shows a large area with now-formed Au nanowires having lengths that range from several hundred nanometers to several micrometers. Figure 4b–e shows four individual, plated structures in which continuous Au nanowires have lengths of 1–2 µm and diameters in the range of 35–60 nm. The diameter of wires varied along their length, from as thin as 23 nm to as thick as 115 nm.

### 2.4. Pd-Seeded and Au-Plated DNA Nanotubes

**Figure 5:**
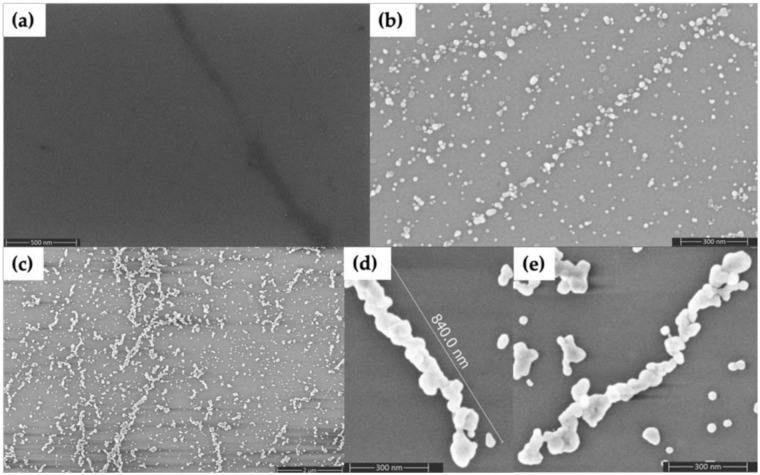
SEM images of DNA nanotubes seeded with Pd and subsequently plated with Au. (**a**) Pd seeded. (**b**) Pd seeded and step 1 Au plated. (**c**) Pd seeded and steps 1 and 2 Au plated. (**d,e**) Individual Pd seeded and steps 1 and 2 Au plated DNA nanotubes.

As an alternative method to Au nanorod seeding, we also tried Pd seeding. Figure 5 shows SEM characterization of the results. The dark feature angling downward in the right side of Figure 5a is a DNA nanotube; the Pd seeds alone provide poor contrast in SEM images. However, the seeds become visible as granular features in Figure 5b after they have been enlarged by the first gold plating step. Importantly, the subsequent, second Au plating step connected these seeds into wire-shaped structures, as seen in Figure 5c–e. The plated structures in Figure 5d,e were in the range of 1–1.5 µm in length and the diameters were in the range of 35–85 nm. Non-specific background particle deposition was greater, variability of nanowire diameter was higher and nanowire continuity was lower, compared to samples formed by seeding with Au nanorods.

### 2.5. Electrical Characterization

**Figure 6:**
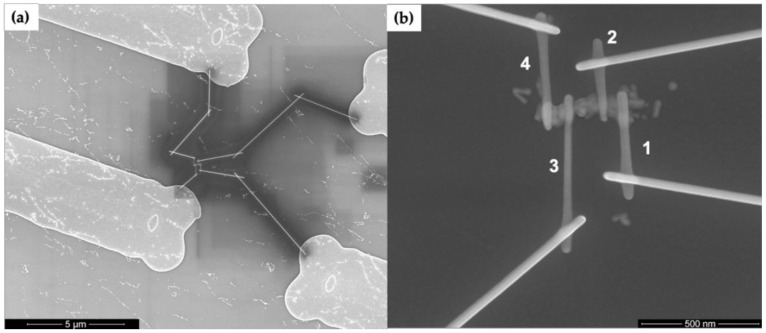
SEM images of electron beam induced deposition (EBID) connections to Au-plated DNA nanotubes seeded with Au nanorods. (**a**) Completed EBID pattern connecting a nanowire to four Au pads. (**b**) Zoom view of the Au plated nanowire in (**a**) with numbered contacts.

We performed electrical measurements to further evaluate the properties and test the continuity of our Au nanowires. Figure 6 shows SEM images of EBID connection to an Au-plated DNA nanotube. Figure 6a shows a wider area view of EBID tungsten patterns connecting the Au nanowire to the arms extending from the Au pads. The EBID patterns have considerable space between each other to limit the effects of overspray of tungsten during writing. Figure 6b displays the four EBID tungsten contacts to a DNA nanotube-templated Au nanowire.

**Figure 7:**
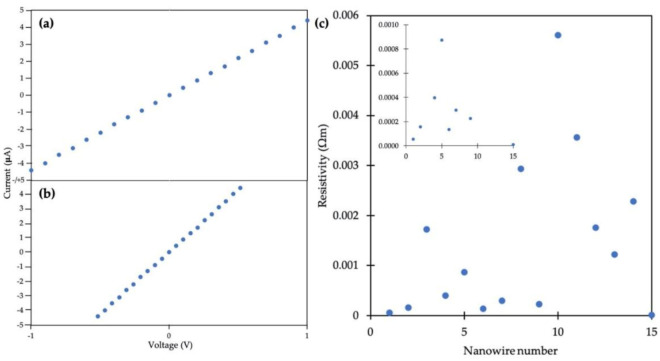
Electrical measurements on a plated DNA nanotube Au nanowire. (**a**) Two point I-V curve. (**b**) Measured voltage drop between inner electrodes during four-point measurement. (**c**) Scatter plot for resistivity values measured. (Inset) Expanded y-axis view from 0 to 0.001 Ωm.

Figure 7a shows a two-point I-V curve from a gold nanowire for input voltages spanning from −1 to +1 V, where leads 1 and 4 in Figure 6b were used for voltage input. Figure 7b shows a four-point I-V measurement for the same gold nanowire where the voltage drop between leads 2 and 3 was measured, along with the current through the wire. For the particular Au nanowire in Figure 7, approximately one half of the input voltage was dropped between leads 2 and 3. The resistance was then calculated by the reciprocal of the slope of the I-V curves in Figure 7. The resistance values for 15 such nanowires out of a total of 41 structures connected via EBID were measured to be between 5 and 167 kΩ.

Resistivity values were determined by using each resistance measured as above, the average distance between leads 2 and 3 and the average nanowire diameter between leads 2 and 3. Figure 7c shows the resistivities determined from fifteen different plated DNA nanotube Au nanowires on five separate Si wafers. Resistivity values ranged from 9.3 × 10^−6^ to 5.6 × 10^−3^ Ωm. The inset in Figure 7c shows an expanded range of resistivity values from 0 to 0.001 Ωm.

## 3. Discussion

The AFM results from Figure 2 indicate that the surface of the oxidized Si wafer has enough DNA nanotubes to form nanowires. The weakly acidic nature of silanol groups that are typically deprotonated allows electrostatic bridging interactions between the negatively charged DNA molecules, the positively charged Mg^2+^ ions and the surface. Additionally, thermally oxidized Si makes it possible to isolate nanowire electrical properties from those of the underlying substrate, while providing a hydrophilic surface for DNA nanotube attachment.

The orientation of Au nanorod-seeded structures shown in Figure 3 was dependent on the arrangement of the DNA nanotubes resulting from their surface deposition. Multiple Au nanorods are visible in some areas of the seeded structures; DNA nanotubes typically flatten from their cylindrical shape and become wide enough to contain multiple seeds because the Au nanorod width is ~10 nm and the DNA nanotube width is ~30 nm. The continuity of the nanowires is more difficult to maintain as nanowires become longer, because the probability increases for having a gap between seeds, which may be removed during processing steps.

We evaluated two different gold plating solutions to fill the gaps between the Au nanorods. When we used a commercial Au plating solution as shown in Figure S2, Au nanorods grew isotropically, leading to Au deposition at the same rate in both the length and width directions. In contrast, when we used the gold plating solution described in Section 4.2.4, we observed that Au nanorods grew anisotropically. We attribute the difference in plating to CTAB, which is not present in the commercial plating solution. When CTAB is present in the plating solution a CTAB monolayer is retained on the sides of the Au nanorods, which inhibits gold deposition on the sides but allows plating on the tips [37]. Different lengths of nanowires (e.g., Figure 4) resulted when DNA nanotubes had different initial lengths or when Au nanorod-seeded structures had larger gaps, which were not filled through the plating process or which appeared due to nanorod liftoff during plating.

Unlike samples seeded with Au nanorods, the Pd-seeded DNA nanotube samples contained many more background particles, as seen in Figure 5. Ionic seeding has poorer selectivity for the desired DNA nanotubes than Au nanorod seeding, as seeding of Pd occurred on all DNA fragments deposited onto the surface. Thus, DNA strands that were not assembled into a tube could also be seeded with Pd, resulting in undesired background metal deposition. In contrast, the background in the Au nanorod-seeded samples was comparatively less than for Pd seeding, so that nonspecific deposition of Au in the plating step was reduced. This lower background for Au nanorod seeding was in part because of one seeding step compared to multiple steps for Pd seeding. In Pd seeding, variability of seed diameter and density was higher than for Au nanorods, as the plating solutions grew the Pd seeds non-selectively and isotropically. Continuity in Pd-seeded nanowires was harder to achieve compared to Au nanorods, as the absence of just one Pd seed could leave a small gap in the nanowire, breaking continuity.

A range of resistivity values was obtained as shown in Figure 7c, which we attribute to differences in the morphology of the wires due to seeding granularity. One conductive structure was considered an outlier and thus omitted from Figure 7c, as it had a resistance of 2500 kΩ, ~15 times greater than the next-largest value measured and a resistivity of 5.95 × 10^−2^ Ωm. This particular structure may have had a short section that was much less conductive in either the nanowire or one of the leads.

EBID can, under some conditions, create “overspray” resulting in conductive material depositing near written wires. Consequently, blank samples were created by connecting EBID tungsten leads (without a nanowire) to contact pads in a configuration like that used for measuring nanowires, as shown in Figure S3a. These blank samples yielded no voltage-dependent current when the input voltage was scanned from 0 to 1 V (Figure S3b); a constant, systematic offset of ~100 pA was observed, which was also seen when the leads were disconnected. As these currents are much lower than those seen in nanowire samples (e.g., Figure 7), we can neglect any current contributions from EBID overspray in our measurements. Figure S4a shows EBID connections to an EBID-written nanowire. We measured the resistance of the EBID-written contact leads in Figure S4a to be ~300 kΩ (Figure S4b). This result confirms that the EBID-written tungsten structures were suitable for conducting electrical measurements since this 300 kΩ resistance was several orders of magnitude lower than the input impedance of our measurement equipment.

Two-point I-V measurements also include the probe, pad, lead and contact resistances, whereas in four-point measurements these extraneous resistances are eliminated. These Au nanowires have resistivities similar to and in some cases smaller than those made by Aryal et al. [38], who measured C-shaped Au nanowires formed on DNA origami with a similar four-point probe technique but obtained resistivities between 4.24 × 10^−5^ and 0.124 Ωm. Our resistivity values reported here are higher than the bulk resistivity of gold (2.44 × 10^−8^ Ωm), probably due to impurities, wire junctions, size effects that cause scattering, presence of surfactant or differences in the processing of nanowires and surfaces.

We successfully obtained four-point I-V curves for 16 out of 41 total structures tested after they were connected by EBID. There were fourteen additional structures where four-point I-V curves failed after showing current in two-point measurements, indicating that nearly three-fourths of these nanowires were conductive at the start of testing. This difference between two-point and four-point results could be caused by gaps in or incomplete contacts from electrode 2 or 3 to the nanowires or by a breakage in one of the leads or the nanowire from resistive heat generated during two-point measurement. Indeed, we confirmed the latter occurrence when in some cases, after running current between leads 1 and 4, the test structure was changed in such a way that it was no longer conductive. For some nanowires where successful four-point I-V curves were not obtained, multiple four-point measurements yielded large variability in the current or voltage drop values, possibly indicative of poor connections (or gaps) between Au-plated structures in the nanowires. If there was a very small gap, electrons could still tunnel across the gap resulting in conductive nanowires but with very high resistances. Another possibility is that the presence of very thin bridging connections between Au nanorods resulted in much higher nanowire resistances.

## 4. Materials and Methods

### 4.1. Chemicals and Materials

Cetyl trimethylammonium bromide (CTAB) (H5882, 98%), hydrogen tetrachloroaurate(III) (HAuCl_4_), sodium borohydride (NaBH_4_), palladium chloride (PdCl_2_), dimethylamine borane (DMAB) complex and 1-methyl-2-pyrrolidinone (NMP) were purchased from Sigma-Aldrich (St. Louis, MO, USA). Ethylenediaminetetraacetic acid (EDTA) was obtained from Life Technologies (Carlsbad, CA, USA). Tris(hydroxymethyl) aminomethane (Tris base) and ascorbic acid were obtained from Fisher Scientific (Fair Lawn, NJ, USA). Acetic acid, hydrochloric acid, magnesium chloride, 4-(2-hydroxyethyl)-1-piperazineethanesulfonic (HEPES) acid and magnesium acetate ((MgAc)_2_·4H_2_O) were obtained from EMD Chemicals (Gibbstown, NJ, USA). Silver nitrate was purchased from Mallinckrodt Chemicals (Philipsburg, NJ, USA). Ammonium chloride was obtained from EM Science (Merck KGaA, Darmstadt, Germany). Gold enhance EM solution was obtained from Nanoprobes (Yaphank, NY, USA). For solution preparation and sample rinsing, water (18.3 MΩ) was generated with a Barnstead EASYpure UV/UF purification system (Dubuque, IA, USA). TAE-Mg^2+^ buffer (10X, pH 8.3) was prepared using 400 mM Tris base, 200 mM acetic acid, 10 mM EDTA and 125 mM (MgAc)_2_·4H_2_O.

### 4.2. Sample Preparation

#### 4.2.1. DNA Nanotube Deposition

DNA nanotube structures were designed and assembled as described by Li and Schulman [39]; Scheme S1 shows the tile sequences. In summary, during self-assembly, nanotube tiles hybridized together to make a lattice, which then generated the hollow DNA nanotubes. After formation the DNA nanotubes were deposited onto plasma-cleaned (Harrick Plasma Asher PDC-32G, 3 min, 18 W), oxidized Si wafers (Polishing Co. of America, Santa Clara, CA, USA) sectioned into 1.5 × 1.5 cm^2^ pieces by adding 5 µL of DNA nanotube solution to the central region and leaving it to adsorb for 15 min in a humidified container to prevent water evaporation. The wafers were then rinsed gently with water and the surfaces were dried under flowing filtered air.

#### 4.2.2. Seeding Au Nanorods on DNA Nanotubes

Gold nanorod synthesis was performed in an adaptation of a published method [37,40]. To obtain larger-diameter nanorods, sodium borohydride concentration was decreased tenfold, as a smaller number of seeds in the Au nanorod growth solution was expected to lead to the production of larger nanorods. The seeding solution (30 µL, Au nanorods in 10X TAE-Mg buffer) was pipetted onto the surface with DNA nanotubes and left to interact for 60 min at room temperature in a humidified container. The samples were rinsed with 4–6 drops of water to clean the surface and to remove nanorods that were not bound to the DNA nanotubes. The samples were dried in a stream of filtered air.

#### 4.2.3. Pd Seeding on DNA Nanotubes

Pd solutions were prepared following a Pd seeding method published previously from our group [23]. In brief, for the activation step, the Pd solution consisting of 1 mM PdCl_2_ and 1 M NH_4_Cl in 10 mM HEPES buffer pH 6.5 was left to adsorb on DNA nanotubes for 2 h in a humidified environment at room temperature. Next, samples were washed with 2–3 drops of water and dried with a stream of air. Then in the reduction step, 40 µL of 40 mM DMAB and 10 µL of 5 mM magnesium chloride were deposited on top of the Pd-treated DNA nanotubes and left to react for 2 min. The reduction step was completed by washing the surface with 2–3 drops of water. The samples were then dried with a stream of air. For repeat seeding, the Pd solution was left to adsorb on DNA nanotubes for 30 min, followed by the same reduction step as above. Multiple Pd seeding steps were performed to increase seed density [23].

#### 4.2.4. Plating Au on Seeded DNA Nanotubes

Electroless plating solution was prepared according to a procedure used previously [40]. Briefly, the plating solution contained 110 mM CTAB, 0.4 mM HAuCl_4_, 0.2 mM AgNO_3_, 0.06% HCl and 1.0 mM ascorbic acid. This solution (70 µL) was pipetted onto wafers with Au-seeded DNA nanotubes and left for 30 min to complete the plating process at 27 °C, after which the samples were rinsed with water and dried with filtered air. For Pd-seeded samples, gold enhance EM solution was applied (70 µL) for 10 min in a humidified chamber and then, the above plating solution (70 µL) was delivered and allowed to react for 30 min at 27 °C. The samples were rinsed with water and dried with filtered air after each treatment of plating solutions.

#### 4.2.5. EBID and Creating Electrical Connections

Photolithography and metal lift-off were used to form square (100 µm) gold contact pads with four extending arms. First, AZ3332 photoresist (Merck kGaA) was deposited and patterned using a SUSS MA 150 contact aligner (Karl Suss America, Waterbury, VT, USA), then developed using AZ300MIF (Merck kGaA). A thermal evaporator was used to deposit a 7 nm chromium adhesion layer, followed by deposition of 50 nm of gold. Lift-off was done by soaking along with shaking (10 min) and sonicating (1 min) in NMP to form the desired contact pads according to previously published procedures [41].

To connect metallized DNA nanotube structures to gold contact pads, tungsten traces (~25 nm wide and using the line dose setting for a height of 1 µm) were patterned using EBID at 5 kV and 0.17 nA in a Helios Nanolab 600 SEM (FEI, Hillsboro, OR, USA) [41,42]. In order to link the thinner tungsten traces to the gold pads, tungsten traces (~25 nm wide and using the line dose setting for a height of 3 µm) were written from the thin traces to the large gold pads.

### 4.3. Characterization

#### 4.3.1. AFM Imaging

DNA nanotubes on wafers were imaged using ‘Peak Force’ tapping mode AFM (MMAFM-2, Bruker, Santa Barbara, CA) with Bruker ScanAsyst automatic image optimization technology and Bruker silicon tips on nitride cantilevers.

#### 4.3.2. SEM Imaging

Seeded and plated DNA nanotube samples were imaged by SEM in ultra-high-resolution mode on a FEI Helios Nanolab 600 or on a Thermo Scientific Verios UC G4 SEM.

#### 4.3.3. Electrical Measurements

A micromanipulator probe station was utilized to connect to the gold pads for I-V studies. A National Instruments DAQ and custom LabVIEW program were used to apply voltages from −1 to +1 V. Current was measured using a DL Instruments 1211 current preamplifier and the output was recorded by the DAQ. Voltage drop values for 4-point probe data were measured simultaneously by a Fluke multimeter (8840A/AF/05, Everett, WA, USA). Measurements were done at room temperature.

## 5. Conclusions

In summary, we used two different methods to fabricate gold-metalized DNA nanotubes, whose electrical properties were subsequently characterized. The first approach involved seeding DNA nanotubes with gold nanorods and connecting them by electroless plating. The second method utilized Pd ionic seeding in a three-step process: Pd activation, Pd reduction to form seeds and electroless plating. Au-seeded and plated structures had continuous lengths that ranged from 1 to 2 µm with diameters of 35-60 nm. Pd-seeded and Au-plated structures had lengths of 1–1.5 µm with diameters of 35–85 nm. We utilized EBID-formed conductive tungsten contacts to study electrical properties of these plated DNA nanotubes. The successfully measured resistance values for 15 nanowires (from a total of 41 structures where electrical characterization was attempted) were between 5 and 167 kΩ. These nanowires could find use as self-assembling interconnects, including 3D structures, that bridge between the molecular- and macro-scale world in future electronic devices.

## Supplementary Materials

Scheme S1: DNA nanotube tile sequences, Figure S1: AFM height characterization, Figure S2: SEM images of Au nanorods seeded and then plated with commercial Au plating solution, Figure S3: Blank experiment to assess overspray or substrate resistance, Figure S4: Control experiment connecting 4 Au pads to an EBID-written nanowire.
