# Supplementary material for: Seeding, Plating and Electrical Characterization of Gold Nanowires Formed on Self-Assembled DNA Nanotubes
Source: Molecules. 2020 Oct 20;25(20):4817. doi: 10.3390/molecules25204817 (PMC7587963; doi:10.3390/molecules25204817)
Supplement: Supplementary file 1 [file molecules-25-04817-s001.pdf]

molecules

Supplementary Materials

Seeding, Plating and Electrical Characterization of Gold Nanowires Formed on Self-Assembled DNA Nanotubes

Dulashani R. Ranasinghe [1], Basu R. Aryal [1], Tyler R. Westover [2], Sisi Jia [3], Robert C. Davis [2], John N. Harb [4], Rebecca Schulman [3] and Adam T. Woolley [1,*]

[1] Department of Chemistry and Biochemistry, Brigham Young University, Provo, UT 84602, USA; dulashani13@gmail.com (D.R.R.); aryalbasu99@gmail.com (B.R.A.)
[2] Department of Physics and Astronomy, Brigham Young University, Provo, UT 84602, USA; tyler.westover13@gmail.com (T.R.W.); davis@byu.edu (R.C.D.)
[3] Johns Hopkins Institute for Nanobiotechnology, Johns Hopkins University, Baltimore, MD 21218, USA; jiasisi1208@outlook.com (S.J.); rschulm3@jhu.edu (R.S.)
[4] Department of Chemical Engineering, Brigham Young University, Provo, UT 84602, USA; john_harb@byu.edu
* Correspondence: awoolley@chem.byu.edu; Tel.: +1-801-422-1701

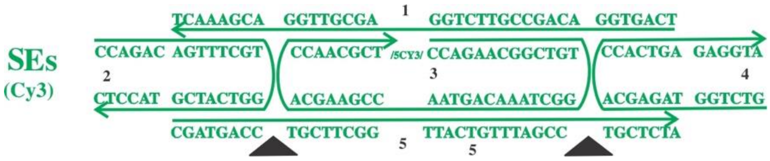

6nt SEs nanotube monomer sequences for SEs tiles within out PEG modification:

SEs_1: TCAGTGGACAGCCGTTCTGGAGCGTTGGACGAAACT

SEs_2: CCAGACAGTTTCGTGGTCATCGTACCTC

SEs_3-5′Cy3: /Cy3/CCAGAACGGCTGTGGCTAAACAGTAACCGAAGCACCAACGCT

SEs_4: GTCTGGTAGAGCACCACTGAGAGGTA

SEs_5: CGATGACCTGCTTCGGTTACTGTTTAGCCTGCTCTA

/Cy3/ denotes Cy3 fluorophore covalently attached to the 5′ end of DNA.

Scheme S1. DNA nanotube tile sequences.

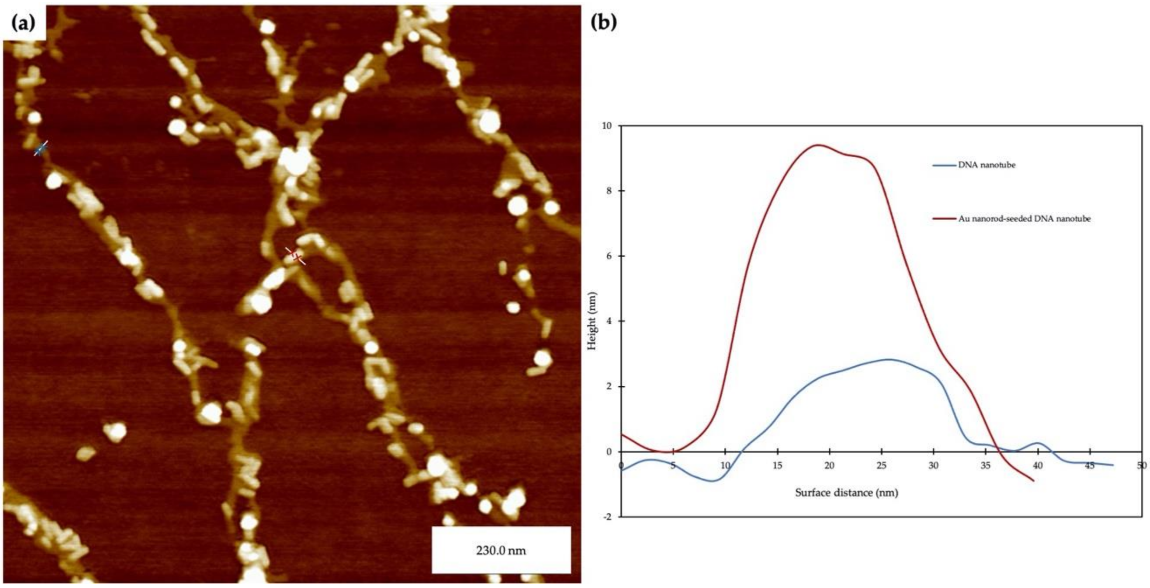

Figure S1. AFM height characterization. (**a**) AFM image of Au nanorods seeded on DNA nanotubes.; height scale: 20 nm (**b**) Height analysis of DNA nanotube (blue) and Au nanorod seeded DNA nanotube (red).

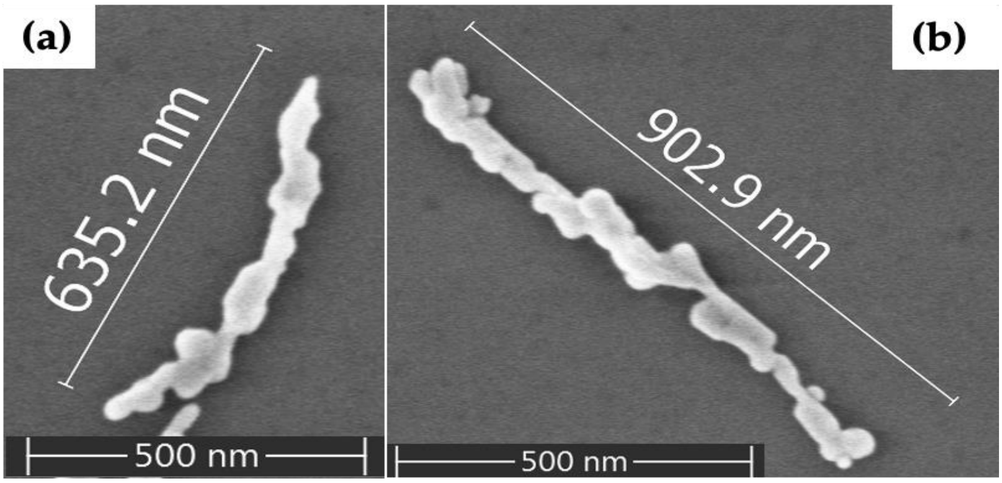

Figure S2. SEM images of Au nanorods seeded and Au plated with commercial plating solution.

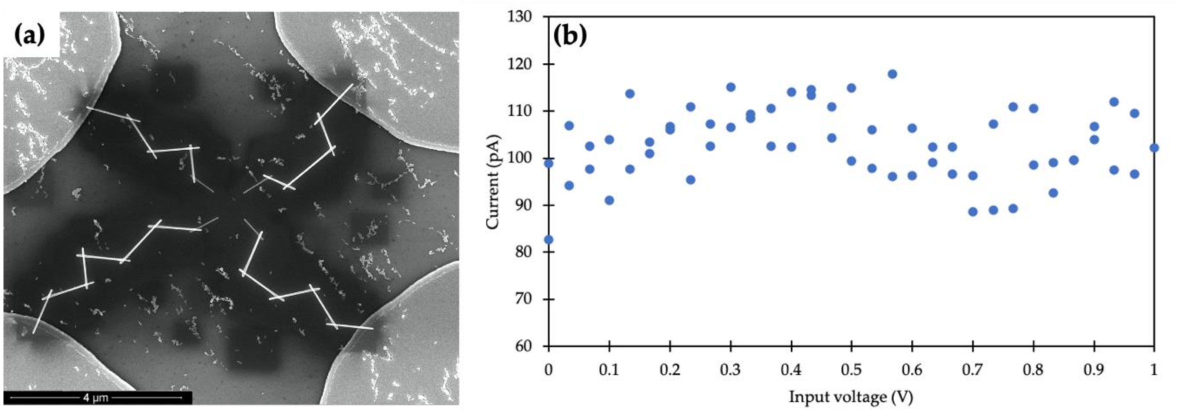

Figure S3. Blank experiment to assess substrate resistance. (**a**) SEM image of EBID connections without a nanowire. (**b**) I-V curve from the setup in (**a**).

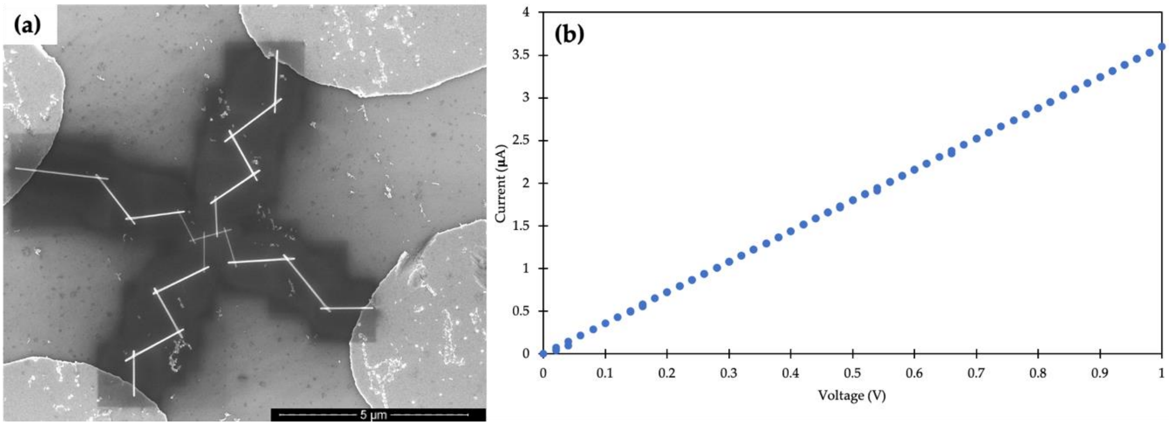

Figure S4. Control experiment connecting 4 Au pads to an EBID-written nanowire. (**a**) SEM image of EBID connections to an EBID-deposited nanowire. (**b**) I-V curve from the setup in (a).
